# What and how do students learn in an interprofessional student-run clinic? An educational framework for team-based care

**DOI:** 10.3402/meo.v21.31900

**Published:** 2016-08-05

**Authors:** Désirée A. Lie, Christopher P. Forest, Anne Walsh, Yvonne Banzali, Kevin Lohenry

**Affiliations:** 1 Department of Family Medicine, Keck School of Medicine, University of Southern California, Los Angeles, CA, USA; 2 Division of Physician Assistant Studies, Department of Family Medicine, Keck School of Medicine, University of Southern California, Los Angeles, CA, USA; 3 Psychology Department, Mount Saint Mary's University, Los Angeles, CA, USA

**Keywords:** student-run clinic, interprofessional education, focus group, theoretical framework, underserved

## Abstract

**Background:**

The student-run clinic (SRC) has the potential to address interprofessional learning among health professions students.

**Purpose:**

To derive a framework for understanding student learning during team-based care provided in an interprofessional SRC serving underserved patients.

**Methods:**

The authors recruited students for a focus group study by purposive sampling and snowballing. They constructed two sets of semi-structured questions for uniprofessional and multiprofessional groups. Sessions were audiotaped, and transcripts were independently coded and adjudicated. Major themes about learning content and processes were extracted. Grounded theory was followed after data synthesis and interpretation to establish a framework for interprofessional learning.

**Results:**

Thirty-six students from four professions (medicine, physician assistant, occupational therapy, and pharmacy) participated in eight uniprofessional groups; 14 students participated in three multiprofessional groups (*N* = 50). Theme saturation was achieved. Six common themes about learning content from uniprofessional groups were role recognition, team-based care appreciation, patient experience, advocacy-/systems-based models, personal skills, and career choices. Occupational therapy students expressed self-advocacy, and medical students expressed humility and self-discovery. Synthesis of themes from all groups suggests a learning continuum that begins with the team huddle and continues with shared patient care and social interactions. Opportunity to observe and interact with other professions *in action* is key to the learning process.

**Discussion:**

Interprofessional SRC participation promotes learning ‘with, from, and about’ each other. Participation challenges misconceptions and sensitizes students to patient experiences, health systems, advocacy, and social responsibility. Learning involves interprofessional interactions in the patient encounter, reinforced by formal and informal communications. Participation is associated with interest in serving the underserved and in primary care careers. The authors proposed a framework for interprofessional learning with implications for optimal learning environments to promote team-based care. Future research is suggested to identify core faculty functions and best settings to advance and enhance student preparation for future collaborative team practice.

The student-run clinic (SRC) is an educational volunteer service activity initiated and coordinated by students under the guidance of licensed faculty, and it offers clinical experiences for students while providing much needed services to the underserved (1–4). Such clinics provide students with clinical experience and exposure to leadership, procedural skills, service learning, and systems-based practice (5–9). SRCs have proliferated, with over 75% of accredited US medical schools reporting more than 208 such clinics in 2014 (10). Through the inclusion of multiple professions, SRCs have been reported as the site of interprofessional learning in the United States (11, 12), Canada (1), and Australia (13), and as sources of access to care for underserved populations (14, 15). Interprofessional education (IPE), defined as students from different health professions ‘learning with, from and about each other’ (16), is considered the basis of interprofessional practice and team-based care, a paradigm shift from the traditional hierarchical health care delivery model intended to improve the efficiency and quality of care (17–20). A recent position paper (21) on the intersection of IPE and collaborative practice stressed that IPE and team-based care are interconnected and that there is a need to apply educational best practices to patient care.

The interprofessional SRC, where students provide direct patient care supervised by attending faculty, is a potential setting to link education to practice. Yet, studies about learning in interprofessional SRCs are limited. One survey reported ‘decline in attitudes’ toward IPE in student volunteers from nursing, medicine, pharmacy, physical therapy, public health, and social work (12). A survey of nursing, medicine, and pharmacy students reported increased student commitment to the underserved (7). Two studies affirmed that students volunteering at an interprofessional SRC (22, 23) valued working with the underserved and with students from other professions. In-depth interviews (9) of 26 student volunteers, leaders, and faculty delineated core student learning as: interprofessional roles, clinic organization, patient factors, health systems, resource management, and systems improvement. These studies address the content (the ‘what’) without exploring in depth the processes (the ‘how’) of learning.

Teaching and learning in interprofessional clinical settings differ from traditional ‘uniprofessional’ clinical rotations in that the focus shifts from the paired preceptor–student relationship to team members learning from one another, facilitated by a preceptor (24). Research that informs the basis of student learning within interprofessional clinical settings is needed to guide effective educational design. To address the literature gap, we conducted a focus group (FG) study of students from four professions who had participated in an interprofessional SRC, using an inductive approach (25) and sensitizing concepts (26). Our research questions were: *What* learning occurs in the interprofessional SRC setting and *How* does learning occur? We aimed to explore both the content and process of learning, and to use grounded theory to derive a framework for understanding how the SRC–IPE experience prepares students for future team-based collaborative practice.

## Methods

### Study setting

Our study was conducted in an urban setting in Los Angeles, California, USA. Our primary care SRC was established in 2011 and includes four health professions: medicine, occupational therapy (OT), pharmacy, and physician assistant (PA). The SRC is located at two health centers serving underserved and uninsured patients. Students sign onto a waitlist to participate in Saturday clinics. Each interprofessional care team consists of one student coordinator, one preclinical medical student, one OT student, one pharmacy student, one preclinical PA student, and, when available, one clinical medical or PA student. Students are overseen by licensed faculty from each profession. Two to three student care teams operate each half-day. Each team cares for one to four patients per half-day with a cycle time of 70–120 min per patient. Each team engages in a team ‘huddle’ (27, 28) before and after the patient encounter, in preparation for presentation to one attending medical faculty. Students either see each patient individually, one profession at a time (i.e., sequentially), or simultaneously as a team of four professions. Students are exposed to both models of patient encounter. In the ‘sequential’ model, students share information about the patient after all encounters are completed to develop a team care plan. In the ‘simultaneous’ model, students generate a care plan immediately after seeing the patient together. After presentation and discussion with attending faculty, a care plan is finalized and implemented by the student team. In our setting, patients are seen for non-emergent, non-urgent chronic illnesses such as diabetes and hypertension, and for preventive care.

*Study Participants* were first-, second-, or third-year health professions students who had participated in SRC sessions in the previous 2 years.

*Recruitment* was done first by email (purposive sampling) using a listserv of students who had worked in the SRC and was supplemented by word of mouth (‘snowballing’) through the student leaders of the SRC (29).

### FG study design

We chose FGs for their ability to elicit group opinions using peer identity to encourage expression of common beliefs and understand consensus or controversy around issues or questions (30, 31). The research team of five comprised clinical and academic faculty representing four health professions (medicine, PA Studies, laboratory science, and psychology). Three researchers had received formal training in IPE. Four have implemented IPE curricula at their institutions and presented IPE programs to faculty and organizations locally and regionally. We used the literature (9, 12, 22, 32–34) as a basis for semi-structured, open-ended questions addressing our research questions. We designed two question guides (Table 1) to address different components of learning (*what* and *how*). To address *what* learning occurred, we first conducted uniprofessional FGs that comprised students of the same profession in each group, to maximize freedom of expression about other professions and to elicit learning content areas that may be profession specific. To address the learning process, we supplemented information from uniprofessional groups by conducting FGs with multiple professions represented. We asked students to compare learning in the SRC setting with learning in their usual rotations and to identify the most effective strategies for promoting interprofessional learning. We applied underlying concepts (16–18, 25, 26) of interprofessional competencies, outcomes of team care, application of learning to other settings, and impact on future practice as guiding principles. Through a process of group discussion and student feedback, we refined and rephrased questions. We aimed to conduct at least two FGs for each profession and two FGs for the multiprofessional groups to achieve theme saturation. FGs lasted an average of 60 min. The groups were moderated by faculty (DL, AW, CF) with extensive experience conducting FGs. Moderators had no role in evaluating participating students. The FGs allowed for an informal atmosphere, natural conversation, opinion differences, and comments from quiet members (29). FGs were conducted in quiet classrooms on campus after class hours. Students received a $10 gift certificate to cover transportation costs.

**Table 1:** Question guides for focus groups for interprofessional student-run clinic, Keck School of Medicine of the University of Southern California, 2016

Key questions	Probes
Uniprofessional groups	
1. Tell us what you learned about another profession that was new or surprising to you, and which profession/s you were most likely to learn something new about.	• Why were you surprised? • How will your learning about the other professions help you in future practice?
2. What were the aspects of team-based care that were improved/worsened compared with care in settings where care is provided by one profession only?	• Why do you think this aspect of care was enhanced/made worse? • Please give an example.
3. Other than interprofessional learning, what else did you learn from the SRC that is likely to impact your future practice?	• Has the experience affected your career choice? Clinical skills? • What did you learn about leadership and management?
4. Share your thoughts about the value of the SRC experience for professional development.	• No probes.
Multiprofessional groups	
1. What are best ways for you to learn to prepare for future practice?	• How do you prepare for future practice? • How do you learn best? • What qualities of precepting help you?
2. How is precepting in the IPE model different or similar from your experience of precepting in your training?	• What did you learn from preceptors? • How is it different from what you learned in your own program rotations?
3. How did the process of care impact your learning in the SRC?	• What did you learn from seeing the patient as a team? • What did you learn from the huddle process?
4. Tell us what you think is the optimal learning environment for IPE.	• No probes.

### Data analysis

FG audio recordings were transcribed, then independently coded by members of the research team representing diverse fields and backgrounds (medicine, PA studies, psychology, education, and laboratory science). We used constant comparison analysis to identify patterns in participants’ perspectives and develop a coding schema (25, 29, 35). Coding occurred in two stages. In the first stage (content analysis and theme categorization), two primary coders (KL and CF) independently identified major themes from text within all transcripts, with reference to our research questions. They generated a common coding schema and then applied the schema to all the transcripts. Their descriptive themes with representative quotes were examined by adjudicating coders (DL, AW, and YB) who also read all transcripts. Separate lists of major themes for the uniprofessional and the multiprofessional groups were constructed. For the uniprofessional groups, differences across professions were described. We expected some overlap in themes from the two types of FGs. Thus, in the second stage (synthesis, analysis, and interpretation), the coders examined the key concepts derived from all themes, to construct an overarching framework that best explains the way students learn in the interprofessional SRC setting. We followed a grounded theory approach (33–35). The two-stage process of data management and interpretation allowed us to remain focused on the research questions while capturing the richness of the raw data from all FGs, to ensure that the theoretical framework that emerged from data synthesis was still grounded in the original text. We used field notes during the FGs to support transcripts and maintained an audit trail. Member checking was performed when the moderator summarized main points and asked participants to confirm and/or modify the summary.

Our study received exempt status approval from the Institutional Review Board (IRB).

## Results

### Participants

For uniprofessional groups, 30 among a master list of 264 eligible students responded to the email invitation. We asked student leaders to recruit additional participants (snowballing). There were 36 students in eight uniprofessional FGs. Fourteen additional students were recruited and they participated in three multiprofessional FGs, for a total of 50 (36+14) students in 11 (8+3) FGs. Student demographics were similar in uniprofessional and multiprofessional groups. Gender was equally represented in medicine (8/14 female); there was a predominance of females (8/9 OT, 9/12 Pharmacy, 14/15 PA students) in the other three professions (Table 2). Students represented both stages of training, preclinical and clinical. All participants reported attending a minimum of two SRC sessions.

**Table 2:** Demographics of students participating in focus groups, Keck School of Medicine of the University of Southern California, 2016, *N=*50

Student profession	Total number in focus groups (*N*)	Age/years *N* for age groups 18–24, 25–29, 30–34, 35–40	Female (*N*)
Medicine	14	9, 5, 0, 0	8
Occupational therapy	9	0, 6, 1, 2	8
Pharmacy	12	9, 3, 0, 0	9
Physician assistant	15	1, 10, 2, 2	14

### FG findings

#### Uniprofessional groups: major themes

The goal of uniprofessional FGs was to explore *what* students learned. Two FGs were conducted for each profession. Theme saturation was achieved for each profession. The two primary coders initially identified 18 and 20 themes, respectively, from their first, independent reading of transcripts. They reduced the list to 10 common themes after discussion to remove redundancy. They confirmed agreement with these themes on their second reading of the transcripts. The adjudicating coders then identified disagreements and overlaps. Face-to-face coder discussion resulted in a final list of six non-overlapping major themes (Table 3). We use letters (student A, B, C, etc.) to represent different students from the respective professions (Medical, OT, Pharmacy, PA).

**Table 3:** Major themes and typical quotes, interprofessional student-run clinic focus groups, Keck School of Medicine of the University of Southern California, 2016

Major themes	Representative quotes, by student profession and letter
Uniprofessional groups (*N=*36)	
Recognition of other professions’ roles and scope of practice	I didn't realize the extent of medication reconciliation that pharmacy can do … how much they know dosing and interactions … how much physicians lean on them. (OT/C) I had no understanding … that PAs played such a large role in managing primary cases as well as … performing surgery. (OT/A) I thought everything musculoskeletal went to PT …. That was one thing I'll use now and in practice: hand issues to OT. (PA/C)
Appreciation of benefits of team-based care	I don't think I'll ever overestimate my profession … asking other professions for help leads to better outcomes in patients. (PA/E) (I learned) that collaboration is for the patient's benefit … the ultimate goal of getting them … healthy. (OT/B) (Patients’) concerns addressed from multiple angles and different people spend time with them, that's a benefit. (Medical/C)
Patient experience of student-run clinic	If the patient has access to people influencing social determinants … you can tackle a problem from all angles …. (PA/A) It can also help the patients, giving them increased access to care because if they were only seen by one provider, they would need to get a referral (Pharmacy/A) The majority (of patients), greater than 90%, would choose team-based care over regular, single provider care …. (Medical/A)
Role of advocacy/systems care	It make sense see how an interprofessional patient-centered medical home could be key to cost reduction, increased quality. (PA/A) … taught me how to be between the student and the preceptor to ensure balance between learning and student engagement … teaches initiative. (Pharmacy/C) I wasn't expecting to take on as much responsibility, and it's been a tremendous learning in leadership. (Medical/A)
Improved leadership and clinical skills	What I gained is (the skill) advocacy, speaking up, explaining the role of OT … fighting for our place in student-run clinic. (OT/A) … the SRC influenced me to go into that areas like the Veterans Administration or underserved clinics. (Pharmacy/A)
Impact on own future career	(The student-run clinic) solidified in me that teaching and team-based care … is a part of me … for the rest of my career. (OT/B) … more time for the patient's story… directed my future career path to go into (primary care) to allow me to do so. (Pharmacy/D) The SRC exposed me to … homeless and impoverished populations … I see myself working with in the future. (Medical/B)
Multiprofessional groups (*N=*14)	
Most valuable learning occurred in the patient encounter	Sitting with the patient taking turns asking questions is effective … we see how professions phrase questions differently (Pharmacy/10) I believe that when we (students) are all in the room together interviewing the patient, it is the best learning situation. (OT/7)
Learning takes place in the huddle, during informal conversations, and during the patient encounter	I've learned from other professions different ways to ask things and the motivation for asking. (Medical/1) Pulling back the lens, seeing it from a wider perspective. Every time you huddle you see the patient in a more holistic way. (OT/7) We definitely learn from the other disciplines when we are around them. (OT/4)
Learning occurs with patients, seniors, peers, and preceptors from other professions	Exposure to students further along in their training facilitates (my) learning. (Medical/5) Informal chitchatting with other professions is helpful. (PA/13) We (have) preceptors who are not OTs and that adds a whole other dimension to what we learn. (OT/4)
Most helpful teaching technique is direct feedback with hands-on practice	I want immediate feedback … instructors that are willing to show first and then have me repeat back are most helpful. (OT/4) (Preceptors are) more helpful if they give me the clinical reasoning behind what they're doing. (OT/3) They show you … then you do it with their guidance. (PA/5)

Medical=medical student; OT=occupational therapy student; Pharmacy=pharmacy student; PA=physician assistant.

Major theme 1: Recognition of other professions’ roles and scope of practice

Students spoke of the specific knowledge gained from working firsthand with colleagues from a different profession. The OT profession emerged as the profession that others learned most about, primarily regarding practice and approach to the patient. For example:

I was surprised by the range of things they (OTs) can do. They have a lot of tools to address different physical and mental issues …. (Medical/A)

Every profession identified some underlying assumption they had about another profession that was challenged or corrected, with examples like:

I've always had this perception that you don't need anyone but physicians for patient care. (Pharmacy/I)

I learned that they (PAs) can write drug orders, do surgery under supervision of a doctor …. (OT/E)

Students went further to identify how future practice behaviors might change, such as:

I've learned a lot about how to communicate better with everyone, and that's definitely going to help me in future. (Pharmacy/A)

Major theme 2: Appreciation of benefits of team-based care

This theme reflected new understanding about care delivered by effective teams whose members communicated well. Students from all professions expressed the need for cohesiveness and the importance of in-person communication, represented by:

I was excited about … all these professions coming together to learn from each other, to provide the best patient care. (OT/E)

Some students commented on the need to establish good communication, leadership, and team process to avoid conflict, for example:

When scopes of practice overlap and there might be a dispute, an expert in this area should take the lead. (Pharmacy/G)

Major theme 3: Patient experience of student-run clinic

Students expressed relief to learn that while patients may be negatively impacted by the additional time needed during a visit, patients also appreciated and perceived a higher quality care received when seen by different professions.

The majority of patients felt that an appropriate amount of time was being spent, which was a welcome surprise (for me). (Medical/A)

Major theme 4: Role of advocacy-/systems-based care

Students related their discovery of systems-based practice in terms of their own roles as leaders and advocates for better healthcare and for improving existing models of care. They also expressed the desire to participate in future policy change (Table 3). For example:

It was the first time I realized the power you have … patients will listen to what you're saying, and you want to make sure that you are doing what's best for them. (PA/C)

Major theme 5: Self-improvement in leadership and clinical skills

Students expressed excitement about developing their skills and gaining competencies earlier than their formal curriculum allowed. Skills included interviewing and physical examination, teaching, leadership, and collaboration. Exposure to the SRC gave students a practical context for applying their skills. Senior students made comments about improving their own teaching skills:

As a senior medical student, to teach clinical skills we've learned … reinforces them. (Medical/H)

… it's taught me (about) being a leader, the servant of all, doing your best and believing in what you do. (OT/F)

Major theme 6: Impact on own future career

Students expressed that the SRC experience led them to consider career paths they may not have considered before, particularly with underserved populations or primary care. Some students saw primary care as the specialty that was most likely to incorporate interprofessional care.

I will start looking for career opportunities with the underserved population or in areas where we have to work with other healthcare providers. (Pharmacy/I)

### Additional themes and descriptions

Unique among OT students, the theme of self-advocacy was dominant, reflected in detailed narratives about patient care that was improved with the participation of the OT student. OT students expressed increased self-confidence about their role in the health care team.

What I really gained is advocacy, speaking up, explaining the role of OT. (OT/A)

… (the SRC) increased my confidence to walk into any facility … approach a physician or nurse and not be intimidated. (OT/A)

In particular, OT (but not the other professions) students noted that their profession's role in primary care was only now being recognized and that this change significantly influenced their own career preferences:

Unlike the other disciplines, primary care is new territory for OT. (OT/C)

Medical students, distinct from the other professions, expressed recognition of their potential power in the patient–doctor relationship and the need to wield that power carefully. For example:

It wasn't until the student-run clinic that I learned how to talk to patients … and communicate in a way respecting of my position of power. (Medical/C)

At the same time, some expressed that the SRC experience taught them humility:

I'll be more open-minded to the idea that the doctor isn't always the expert, they don't always have to be in charge. (Medical/C)

In addition, within the uniprofessional groups, students provided descriptions that distinguished between learning within and outside the patient encounter. The pre-huddle, for example, was lauded for its preparation for teambuilding and effective team-based care:

These huddle times improve patient care … communicating face-to-face is really important. (Pharmacy/E)

Within the patient encounter, students commented on the importance of seeing (vs. hearing about) and experiencing firsthand how other professions function:

I was surprised by the difference in perspective of a medical or OT or PA student, for example, on whether a patient was taking their medication. (Pharmacy/G)

Students spoke of the benefits of the post-encounter huddle as a form of checks and balances against errors, as well as facilitating mutual appreciation of one another's scope and range of practice.

With each student that went in to talk with a patient and reported to their team, the story continued to unravel, and our overall picture became much clearer. (PA/F)

Another profession might have a different scope of practice, so they're able to put the puzzle together better. (OT/E)

Students even commented on positive aspects of the precepting process and demonstrated appreciation of the importance of good teaching.

Being a preceptor and giving back to your profession is fundamental … seeing it in action makes me appreciate that (teaching) will be a part of my future career. (PA/F)

#### Multiprofessional groups: major themes

We conducted three multiprofessional FGs with 14 students, each represented by three to four professions. We identified four new themes (Table 3) addressing the ‘how, where, when, and who’ of learning. We use numbers (student 1, 2, 3, etc.) to represent different students from each profession for the multiprofessional FGs. Students emphasized the role of *direct observation* of other professions during the patient encounter as a primary contributor which stimulated reflection on how other professions reason and think. They expressed a preference for the ‘simultaneous’ over the ‘sequential’ model of seeing the patient (for example, ‘I believe that when we are all *in the room together* interviewing the patient it is the best learning situation’, OT/7). Students emphasized that direct observation and interaction in team huddles and informal socialization reinforced and consolidated learning. They remarked on how this ‘wider lens’ on providing care resulted in more ‘holistic’ and ‘patient-centered’ care. Students explicitly noted that, in addition to learning from preceptors, they learned from patients, their seniors, and peers from other professions.

#### Combined key concepts and framework

**Fig. 1:**
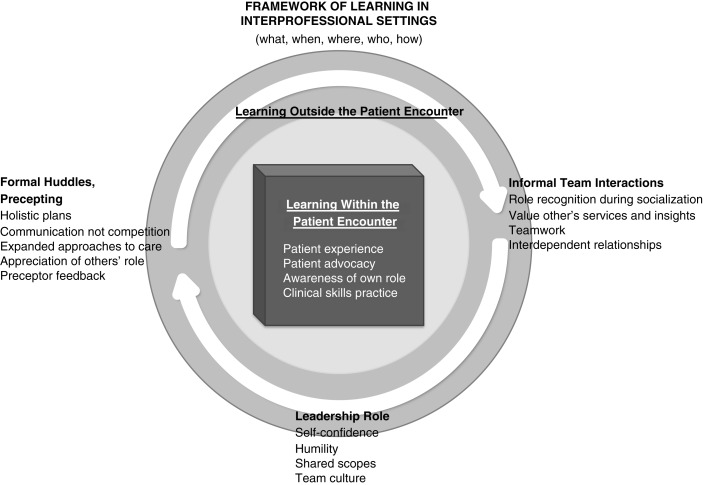
Proposed framework for learning in interprofessional student-run clinic environment.

Themes representing the content of what students learned were well-defined (Table 3), while themes about process of learning separated into *when, where, how*, and *from whom* learning occurred. Concepts of *how* students learned (addressing our second research question) emerged in themes from both uniprofessional and multiprofessional FGs. We synthesized information from all sources to develop a coherent framework that captures the student experience of learning in the IPE–SRC setting (36–39). We propose in this model (Fig. 1) that learning occurs both within and outside (*where*) the patient encounter. Within the patient encounter, students are focused on the task of eliciting information to develop a plan (*how*) to present to the attending. When functioning within a team, they become more conscious of their own individual roles and responsibilities as well as that of other professions. Being in the presence of the underserved patient (*when*) among others promotes professional formation (*what*) in the values of compassion, advocacy, and courage (40). For example, students express empathy (*what*) for the barriers (language, cost, and education) underserved patients face in obtaining services.

Outside the patient encounter****, interprofessional interactions generate a different kind of learning. In the structured and *formal* huddle (*when*), students value team communication over competition (*what*) as a system of checks and balances for ensuring holistic patient care. Students recognize that they learn from other professions, seniors, preceptors, and the patient (*who*). They develop a deeper appreciation for each other's services (e.g., pharmacist's practical knowledge about pill sizes and OT's practical experience with patient education). Further socialization occurs in *informal* communications (*when* and *who*) unrelated to a specific patient's care, in which students appreciate each other's functions and roles (e.g., by asking about one another's training). This dynamic reflects higher-level networking among professions (41).

## Discussion

We conducted a study to examine learning content and processes associated with exposure to volunteer sessions at an urban primary care SRC. Our themes about *what* was learned affirmed and extended previous findings (9, 22). We identified additional themes of gain in own skills and impact on future career, specifically primary care and working with underserved patients. By separating professions, we augmented previous studies and identified new themes unique to OT (self-efficacy and self-advocacy) and medicine (humility and responsible leadership). Medical students alluded to the need to respect other professions and avoid arrogance and dominance. We did not identify any themes related to power and hierarchy reported in other studies (42–44). We speculate that this absence may reflect collegiality among students who have not yet been exposed to the culture of medical dominance in practice and underscores the importance of early exposure to other professions to build mutually respectful collaboration (45–47).

Our study revealed *how* students learn (Fig. 1), shedding light on the dual need for optimal facilitation from faculty preceptors and appropriate private space for student collaboration. Direct simultaneous (vs. sequential) patient care and immersion with other professions emerge as key contributors to interprofessional learning. The process of collaboration, whether in team huddles, informal socialization, assessing patients’ needs, or presenting to the attending faculty, provides opportunities to ‘stand in the shoes of other professions’ and to deepen appreciation for others’ roles. Learning occurs in a continuum around each patient's care, from the time of the pre-encounter huddle to informal socialization and reflection after the formal presentation. The findings emphasize the primacy of face-to-face interactions and shared patient care over electronic or telephonic communication as a model of team-based care. Our study complements and extends reports about service learning (40, 48) and community-based programs (49, 50) that describe interprofessional learning as ‘transformative’, when students from different professions work with clients from underserved settings with a common goal to improve patient care. The underserved setting appears to deepen awareness of team processes, allowing students to articulate ‘beliefs, emotions, and behaviors related to interprofessional teamwork’ (48).

Our findings also suggest that students develop interdependent relationships while engaged in interprofessional socialization. For example, students given leadership opportunities express both increased self-confidence and humility. The student leaders’ reflections on their role support the concept of ‘team-learning leaders’ as a step in adapting to an interprofessional culture (41, 43). Among medical students in particular, the interprofessional leadership role may serve to minimize competition and diffuse the culture of medical dominance found in many academic clinical settings (41, 43).

Our study has several strengths. We examined the questions of learning content and process using two separate guides, which allowed in-depth exploration. We had diverse representation of students, professions, and researchers. We achieved theme saturation for both uniprofessional and multiprofessional groups and used a rigorous coding process for data interpretation. Finally, we applied a robust data synthesis approach to propose our learning model. There are also limitations. Our study was conducted at a single institution; other professions such as nursing, social work, and dentistry were not represented. Findings in the outpatient underserved primary care setting may not apply to other settings, and our findings regarding career preferences may be reflective of self-selection into the SRC experience.

## Conclusions

Participation in interprofessional SRC sessions teaches role understanding, patient advocacy, and team-based care, and increases the likelihood of considering a career in primary care and/or service to the underserved. Direct observation of other professionals at work during patient care is essential. Students identify a continuum of learning from the pre- to post-patient encounter team huddle and the preceptor presentation to informal social interactions. The IPE setting offers unique team-based practice opportunities not available in traditional uniprofessional rotations. Educators need to remain aware of the teaching environment and create physical spaces that allow student teams to huddle and see patients together. Studies could be conducted in other settings to evaluate the transferability of our proposed model. Future research is needed to identify critical activities that contribute to interprofessional learning, and characteristics of faculty precepting that build team skills, with the ultimate goal of best preparing students for future collaborative practice.
